# A retrospective longitudinal observational study of natural history of ESBL/MDR gram negative organisms in patients hospitalized in a tertiary hospital

**DOI:** 10.1017/ash.2022.157

**Published:** 2022-05-16

**Authors:** Shikha Vasudeva, Anthony Baffoe-Bonnie

## Abstract

**Background:** The IDSA recommends that when determining empiric treatment for a given patient, clinicians should consider previous organisms and associated antibiotic susceptibility data in the prior 6 months when multidrug-resistant/extended-spectrum β-lactamase gram-negative rods (MDR /ESBL GNR) is suspected. The presence of MDR GN in cultures on previous hospitalization or in surveillance cultures leads to use of broader-spectrum empiric antibiotic in subsequent admissions. We sought to determine whether the presence of an MDRO in 1 hospital admission dictates the need for broader-spectrum antibiotic use in subsequent hospitalizations when a different site is considered the source of infection. **Methods:** The study was conducted at a 700-bed tertiary-care level I trauma center. Infection control records were reviewed for patients aged 21–99 years admitted between July 1, 2013, through January 31, 2014, who had any ESBL or MDR GNR at any site including surveillance culture. The charts were surveyed until January 31, 2016, for ESBL or MDR gram-negative organisms at any site during subsequent hospitalizations. The CDC definition of MDR/ESBL was applied. **Results:** Of the 50 people followed, 34 patients (68%) had ESBL/MDR recovered at a single sentinel site, 6 patients (12%) had ESBL/MDR in multiple sites, and in 10 patients (20%) had ESBL/MDR only in surveillance cultures during their primary hospitalization. In 34 patients with a single sentinel site MDRO/ESBL in their primary hospitalization, 16 (47%) were identified in urine, 13 (38%) were identified by bronchoalveolar lavage (lung), 4 (12%) were identified on skin or MS, and 1 (3%) was identified at another site. When lung and urine were the sole sites of recovery of MDRO/ESBL in primary hospitalization, respectively, 3 patients (23%) and 2 patients (12.5%) subsequently developed MDROs in a secondary site on subsequent admissions. Overall, 10 bacteremia episodes occurred in 8 patients among 189 total admissions. MDRO/ESBL *Klebsiella* spp were identified in the cultures of 5 patients (50%), MDRO *Acinetobacter* spp were identified in the cultures of 3 patients (30%), and ESBL *E. coli* was identified in the cultures of 2 patients (20%). The organism causing bacteremia was present at a different sites during the same admission in only 3 (30%) of these cases: 2 were cultured from urine and 1 was cultured from a pulmonary source. **Conclusions:** The presence of MDRO at 1 site in a previous hospitalization may not be sufficient evidence to justify very broad antibiotics for patients during subsequent admissions, especially when another site is considered the source of infection. More studies are needed on natural history of MDRO in different patient populations with different risk factors.

**Funding:** None

**Disclosures:** None

